# The Gibco^™^ CTS^™^ Rotea^™^ system story—a case study of industry-academia collaboration

**DOI:** 10.1038/s41434-021-00266-6

**Published:** 2021-06-10

**Authors:** Anqi Li, David James, Rebecca Lim

**Affiliations:** 1 The Ritchie Centre, Hudson Institute of Medical Research, Clayton, VIC Australia; 2 Department of Obstetrics and Gynaecology, Monash University, Clayton, VIC Australia; 3 Scinogy Pty Ltd, Box Hill, VIC Australia

**Keywords:** Stem-cell therapies, Cell therapies

## Abstract

The Gibco^™^ CTS^™^ Rotea^™^ Counterflow Centrifugation System is an automated cell processing device developed for manufacturing cell therapy products. The developer (Scinogy Pty Ltd) collaborated with Thermo Fisher Scientific to successfully launch the product in late 2020, completing product development from concept to international sales in <3years. This article describes the origin story of the Rotea system and how a chance meeting between a co-inventor of the Rotea system and an academic cell biologist took the invention from a garage workshop to the world stage. We describe the contribution of academic research to the innovation value chain and importance of academic institutions being industry-ready to support such collaborations.

## Background

There is no doubt that the market approval of the first chimeric antigen receptor T (CAR-T) cell therapy product heralded the dawn of a new age for cell and gene therapies (CGT). It was proof positive that a CGT product could be cost-effective and commercially viable [1–3]. Unsurprisingly, this has coincided with a rapid growth in the market with substantial increases in the number of clinical trials into this field [4–6]. One of the considerable knock-on effects of growth in this sector has been an increased interest in automated solutions for product manufacturing [7].

Academia-industry collaboration plays an important role in cell and gene therapy development, particularly in resolving some of the manufacturing challenges [8, 9]. Historically, academics provide ideas and initial findings but rarely do they continue to participate in product development. Nor are they typically responsible for providing the infrastructure for product manufacturing [8, 9]. Here, we present a case study of academia-industry collaboration where initial findings and prototype development were provided by the industry partner, Scinogy Pty Ltd. In this instance, the academic partners were involved in product testing and validation studies, without ownership of the underlying intellectual property and benefitted in non-financial ways.

## The Rotea system: the origin story

**Fig. 1:**
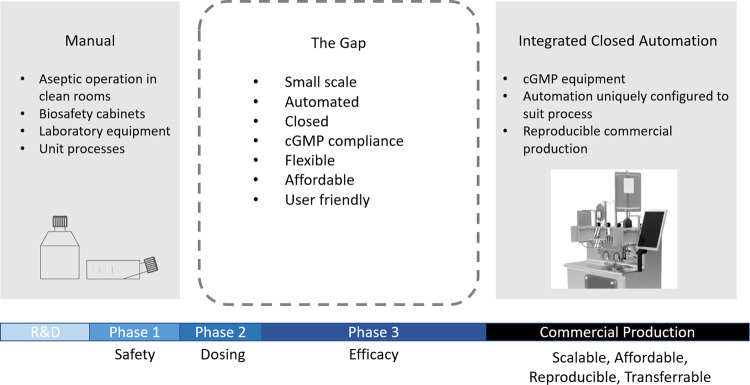
Identification of the gaps in cell manufacturing processes. Early phases of process development typically involve open, manual processing steps which can be highly variable between operators and rely on aseptic techniques, biological cabinets and cleanrooms to maintain sterility. While automated processes are not often considered until commercial scale manufacturing is required, where automated solutions are often created specifically for the process and flexibility is traded off.

It had already been proven that manual, open processes were extremely difficult to scale for clinical production [10]. However, the alternative of developing custom manufacturing systems was expensive, time consuming and risky, especially when the clinical outcome was uncertain [11]. It is also desirable to have a flexible manufacturing process comprising of unit devices (i.e. bioreactors, concentration, fill and finish, etc.) that can be optimised, and ultimately qualified and integrated where practical for clinical production, connected by software for GMP (Good Manufacturing Practice) compliant manufacturing and batch record generation [12] (Fig. 1).

With more than a decade consulting to the industry, it became obvious to the founders of Scinogy that cell and gene therapy companies needed tools that were designed to suit the specific needs of the products from research through to commercial production [13]. Specifically, they sought to develop a product that was affordable for small scale, early-stage product development and academic users, but also had the capacity to accommodate larger commercial scale production. One of the key technology gaps was automated washing and buffer exchange of autologous cell products where smaller input volume (<1 L) and output volumes (5–50 mL) were commonplace (Fig. 1) [14].

In November 2016, the Rotea system was conceptualised as a convergence of thinking by the inventors, while writing an article on cell therapy manufacturing and listening to presenters at the Cell Therapy Manufacturing & Gene Therapy Congress in Europe [13].

The Scinogy team subsequently developed the bench top counterflow centrifugation device that utilises enclosed, single-use kits amenable to the manufacturing scale of autologous cell products. The first patents were filed just prior to a serendipitous meeting on the Nozomi shinkansen (bullet) train hurtling from Yokohama to Kobe, where David James (CEO Scinogy Pty Ltd) met Associate Professor Rebecca Lim (Scientific Director, Cell Therapies & Regenerative Medicine Platform, Hudson Institute of Medical Research). Until this point, the prototype device had only ever been tested using beads and in the setting of the humble garage workshop. The time had come to test the Rotea system on live cells. As a young start-up company comprising solely of engineers, it was clear that this stage of prototype testing necessitated access to a cell culture facility and of course, access to cells and preferably a cell biologist with extensive experience to cell manufacturing for clinical trials. This chance meeting led to the collection of initial datasets sufficient to support the first public showcasing of the Rotea system at the Phacilitate conference in Miami in January 2018.

**Fig. 2:**
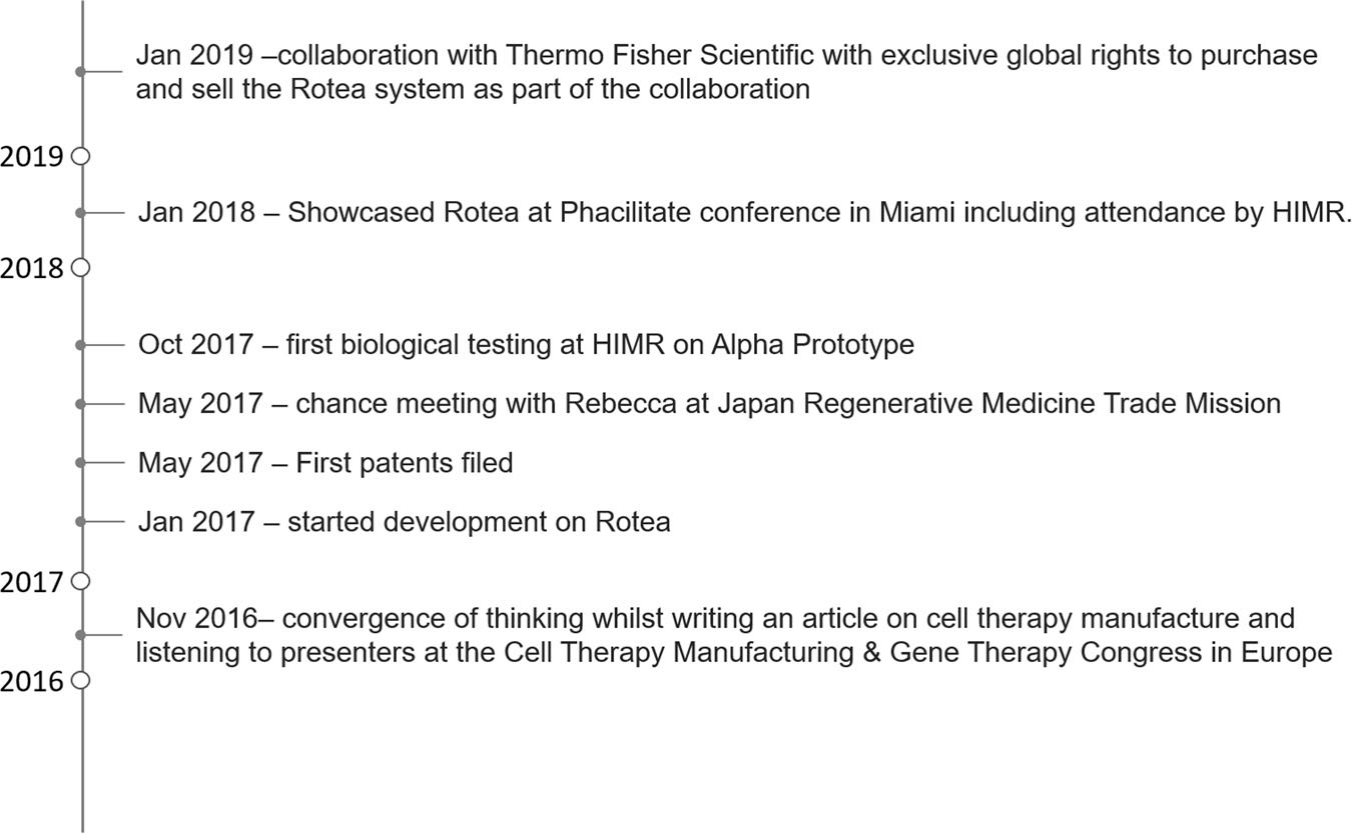
Origin story or the Rotea system. Timeline summarising the developmental milestones of the Rotea system.

It was through this chance meeting that the assessment of the initial protype and subsequent process development on the Alpha prototype instrument became the foundation of a PhD project jointly supported by Scinogy and Hudson Institute of Medical Research. The ability of the Rotea system to perform buffer exchange and volume reduction was published in in the Journal of Visualised Experiments [15]. Here, we published the first working protocols on the Rotea system for enclosed, automated cell washing, buffer exchange and volume reduction. This academic publication utilised human umbilical cord tissue derived mesenchymal stem/stromal cells (MSC) and Jurkat cells to demonstrate that the Rotea system was capable of rapid cell processing with extremely high cell recoveries while retaining cell function [15]. This observation was instrumental to the collaboration between Scinogy and Thermo Fisher Scientific regarding the Rotea system and Thermo Fisher Scientific securing exclusive global rights to purchase and sell the Rotea system in January 2019 (Fig. 2).

## When academic involvement adds value

Initially, one may question the inherent value of academic involvement in this project. At face value, it appeared that the Rotea system prototype was ready for prime time with patents filed and much of the hardware development complete. The Scinogy team had already showed that the Rotea system was efficient in buffer exchange, volume reduction and elutriation of beads into predetermined volumes [16], so why complicate matters by involving an external team? The value of this interaction laid in the ability of the academic team to provide a variety of common cell manufacturing input material for prototype testing. Through this collaboration, the Rotea system prototype was taken through its paces using expanded MSC [15, 17], immortalised Jurkat cells [15, 17], primary amniotic epithelial cells [18], expanded T cells and cryopreserved leukapheresis products.

The jointly supported PhD project was one of the key elements in this collaboration. The scholarship and majority of the project expenses were supported by government grants. The involvement of an enthusiastic, industry-minded PhD candidate further precipitated the design of key experiments aligned with the principles of Quality-by-Design, in an effort to identify critical processing parameters for common cell therapy products *(manuscript under review)*. The involvement of autonomous academics enabled independent, unbiased collection of diverse data that addressed decision making criteria by parties interested in the technology. Scinogy was then able to use these data to demonstrate the value of the Rotea system since their prototype had now been tested in ‘real world settings’. These early unpublished experiments were to demonstrate the ability of Rotea system to address common pain points for the cell manufacturing industry, including the removal of red blood cells without necessitating density gradient reagents, removal of dead cells from cryopreserved input material and rapid volume reduction for cultured cells. That a proportion of this data was published in a peer-reviewed journal and presented at annual scientific meetings of learned societies such as the International Society for Cell and Gene Therapy [17, 19], added credence to the industry-academia collaboration, while creating value for the PhD candidate and providing valuable independently sourced data for consideration during licensing deal discussions.

## Creating fertile soil for industry-academia collaborations

It is important to recognise the role of the environment for nurturing such collaborations. In this instance, the Hudson Institute of Medical Research facilitated the academia-industry collaboration by providing an office space for the Scinogy team during the early days of product testing. In addition, the institute did not make claims on intellectual property—recognising that the data collected would support the development of the Rotea system while also enabling the publication of novel research findings by Hudson researchers.

The Hudson Institute being co-located with Victoria’s largest public healthcare provider, Monash Health, and a prestigious university, Monash University, on a large medical research precinct, was extremely advantageous for Scinogy Pty Ltd. Furthermore, the numerous cell therapy developments [20, 21] and clinical trials [22–25] underway on the precinct meant that Scinogy received direct, continuous and vital feedback from users of the Rotea system.

Unsurprisingly, Hudson Institute hosted a number of visits by parties expressing interest in the licensing of Rotea system and were equally interested in watching it in use. Following the execution of the agreement with Thermo Fisher Scientific, Hudson Institute saw increased traffic by potential buyers of the Rotea system from the Asia-Pacific region, who were predictably interested to understand its utility by current users of the device. This arrangement was beneficial to both the Hudson Institute and Thermo Fisher Scientific. The Hudson Institute was relatively young and was still growing its global reputation. Thermo Fisher Scientific was able to use the facilities of the Hudson Institute to demonstrate the instrument using real cells. Needless to say, this was also beneficial to the academic team which has since received global recognition for their expertise in the Rotea system processing (Technology Showcases at Phacilitate 2019 and ISCT 2019).

## Conclusions

Academics are an important part of the R&D value chain and clinical translation of discoveries can take many forms. The role of academics in the clinical translation process can extend beyond the early discovery stage, and perhaps the apparent rarity of such collaborations is due to the preconceived notions around prescribed roles of contributors at different parts of the R&D value chain. This case study presents an example where the intellectual property does not have to have arisen from the academics for them to make significant contributions to the innovation value chain. Indeed, the cell and gene therapy sector is one that continues to involve academics in product manufacturing throughout the phases of product development [8]. Academic institutions should therefore put measures in place that facilitate such interactions when such opportunities arise.

To quote the Roman philosopher, Seneca, *“Luck is what happens when preparation meets opportunity”* and with the right preparation, all parties may come away feeling incredibly lucky to have been involved.

## References

[1] Fiorenza S, Ritchie DS, Ramsey SD, Turtle CJ, Roth JA (2020). Value and affordability of CAR T-cell therapy in the United States. Bone Marrow Transplant.

[2] Furzer J, Gupta S, Nathan PC, Schechter T, Pole JD, Krueger J (2020). Cost-effectiveness of Tisagenlecleucel vs Standard Care in High-risk Relapsed Pediatric Acute Lymphoblastic Leukemia in Canada. JAMA Oncol.

[3] Jorgensen J, Hanna E, Kefalas P (2020). Outcomes-based reimbursement for gene therapies in practice: the experience of recently launched CAR-T cell therapies in major European countries. J Mark Access Health Policy.

[4] Seimetz D, Heller K, Richter J (2019). Approval of First CAR-Ts: have we Solved all Hurdles for ATMPs?. Cell Med.

[5] Tang J, Hubbard-Lucey VM, Pearce L, O’Donnell-Tormey J, Shalabi A (2018). The global landscape of cancer cell therapy. Nat Rev Drug Discov.

[6] ARM Annual Report & Sector Year in Review: 2019. https://alliancerm.org/sector-report/2019-annual-report/. Accessed on 25 Apr 2021.

[7] Vormittag P, Gunn R, Ghorashian S, Veraitch FS (2018). A guide to manufacturing CAR T cell therapies. Curr Opin Biotechnol.

[8] Hildebrandt M (2020). Horses for courses: an approach to the qualification of clinical trial sites and investigators in ATMPs. Drug Discov Today.

[9] Pearce KF, Hildebrandt M, Greinix H, Scheding S, Koehl U, Worel N (2014). Regulation of advanced therapy medicinal products in Europe and the role of academia. Cytotherapy..

[10] Better M, Chiruvolu V, Sabatino M (2017). Overcoming Challenges for Engineered Autologous T Cell Therapies. Cell Gene Ther Insights.

[11] Rafiq QA, Thomas RJ (2016). The evolving role of automation in process development & manufacture of cell & gene-based therapies. Cell Gene Ther Insights.

[12] Smith D, Heathman TRJ, Klarer A, LeBlon C, Tada Y, Hampson B (2019). Towards Automated Manufacturing for Cell Therapies. Curr Hematol Malig Rep.

[13] James D (2017). How short-term gain can lead to long-term pain. Cell Gene Ther Insights.

[14] Scibona E, Morbidelli M (2019). Expansion processes for cell-based therapies. Biotechnol Adv.

[15] Li A, Wilson S, Fitzpatrick I, Barabadi M, Chan ST, Krause M, et al. Automated Counterflow Centrifugal System for Small-Scale Cell Processing. J Vis Exp. 2019. 10.3791/60423. 10.3791/60423 31885382

[16] CTS Rotea bead kit demo https://www.thermofisher.com/au/en/home/clinical/cell-gene-therapy/manufacturing-solutions/rotea-counterflow-centrifugation-system/resources.html.

[17] Li A, Kusuma G, James D, Lim R (2020). Design of Experiment (DoE) approach to identify critical parameters in a counterflow centrifugation system. Cytotherapy..

[18] Lim R, Li A, Kusuma G, Chan S, McPhee G, Fitzpatrick I (2019). Enabling clinical trials in an academic GMP setting through use of closed, semi-automated manufacturing of allogeneic amniotic epithelial cells. Cytotherapy..

[19] Li A, Wilson S, Fitzpatrick I, Chan ST, Barabadi M, Kusuma GD, et al. Evaluating automated buffer exchange protocols using Rotea^™^ counterflow centrifuge. Cytotherapy. 2019;21:S40.

[20] Malhotra A, Castillo-Melendez M, Allison BJ, Sutherland AE, Nitsos I, Pham Y (2020). Neurovascular effects of umbilical cord blood-derived stem cells in growth-restricted newborn lambs: UCBCs for perinatal brain injury. Stem Cell Res Ther.

[21] Eggenhuizen PJ, Ooi JD (2020). T cell receptor transduction onto regulatory T cells to treat autoimmune disease. Cytotherapy.

[22] Lim R, Hodge A, Moore G, Wallace EM, Sievert W (2017). A Pilot Study Evaluating the Safety of Intravenously Administered Human Amnion Epithelial Cells for the Treatment of Hepatic Fibrosis. Front Pharmacol.

[23] Lim R, Malhotra A, Tan J, Chan ST, Lau S, Zhu D (2018). First-In-Human Administration of Allogeneic Amnion Cells in Premature Infants With Bronchopulmonary Dysplasia: a safety study. Stem Cells Transl Med.

[24] Phan TG, Ma H, Lim R, Sobey CG, Wallace EM (2018). Phase 1 Trial of Amnion Cell Therapy for Ischemic Stroke. Front Neurol.

[25] Baker EK, Malhotra A, Lim R, Jacobs SE, Hooper SB, Davis PG (2019). Human amnion cells for the prevention of bronchopulmonary dysplasia: a protocol for a phase I dose escalation study. BMJ Open.

